# Association of systemic immune-inflammatory index with all-cause and cancer mortality in Americans aged 60 years and older

**DOI:** 10.3389/fragi.2025.1502746

**Published:** 2025-03-10

**Authors:** Wangfeng Lu, Yuliang Gong, Lei Liu, Yonghong Zhang, Xiaojian Tian, Huanxian Liu

**Affiliations:** ^1^ Department of Gastrointestinal Surgery, Shangluo Central Hospital, Shangluo, Shanxi, China; ^2^ Department of Neurology, First Medical Center of Chinese PLA General Hospital, Beijing, China

**Keywords:** SII, mortality, cancer, inflammation, NHANES

## Abstract

**Background:**

This research delved into the association between the systemic immune-inflammatory index (SII) and both all-cause and cancer-specific mortality among individuals aged 60 years and above in the United States during the period from 1999 to 2018, with follow-up extending until 31 December 2019. The data utilized was sourced from 4295 population-based participants in the National Health and Nutrition Examination Survey (NHANES).

**Methods:**

To analyze the relationship between SII and mortality, the study employed Cox proportional-risk models, restricted cubic spline curves, survival curves, and subgroup analyses.

**Results:**

The average age of the participants was 70.7 (±7.6) years, the median follow-up duration was 131.7 (±59.8) months, and the all-cause mortality rate stood at 50.5%. Findings from the Cox regression model indicated that, after adjusting for covariates, SII was significantly and linearly related to all-cause mortality (hazard ratio HR = 1.31, 95% confidence interval CI = 1.15–1.48). Moreover, the relationship between SII and cancer mortality exhibited a U-shaped pattern. Results from the survival curves suggested that a higher SII was associated with an augmented risk of both all-cause mortality and cancer mortality.

**Conclusion:**

There is a significant association between higher SII levels and increased risk of all-cause and cancer-specific mortality in the US population aged 60 years and older.

## Introduction

Chronic diseases and cancers have emerged as the primary culprits behind the high mortality rates among the elderly (Silva et al., 2023). As society steadily marches towards an ageing demographic, the incidence of these conditions among the senior population has witnessed an explosive upsurge. Projections indicate that by 2050, a full 20% of the global population will be 60 years old or older. Concomitantly, there will be a substantial hike in the incidence and mortality associated with age-related chronic diseases. Notably, the number of newly diagnosed cancer cases is anticipated to skyrocket to 35 million, and the death toll from cancer is projected to climb to 10 million (Fane et al., 2020; Bray et al., 2022). This not only poses a grave threat to the well-being of the elderly but also exerts a colossal burden on the economy and healthcare systems.

Inflammation is widely regarded as a pivotal endogenous factor in the ageing process, playing a crucial role in the development of chronic diseases and cancer (Li C. et al., 2023). SII serves as a key indicator of the body’s immune-inflammatory status. It is calculated by measuring neutrophils, platelets, and lymphocytes in peripheral blood samples (Feng et al., 2022). Clinically, it has been extensively employed to prognosticate the outcomes of various diseases, including cancer, cardiovascular and cerebrovascular diseases, diabetes mellitus, and sarcopenia (Tian et al., 2022; Ye et al., 2022; Han et al., 2023; Xie et al., 2024).

There is a significant increase in the incidence of chronic diseases and cancers among people over 60 years of age (Thomas et al., 2020; Fane et al., 2020). However, within the U.S. population aged 60 and older, the association between the SII and both all-cause and cancer mortality remains relatively under-investigated. Therefore, in this study, we harnessed data from the National Health and Nutrition Examination Survey (NHANES) to thoroughly explore the relationship between the systemic immune-inflammatory status and mortality outcomes in this specific demographic.

## Materials and methods

### Data sources

The NHANES is a nationally representative survey executed by the National Center for Health Statistics (NCHS). It utilizes stratified, multistage probability cluster sampling to evaluate the health and nutritional status of the non-institutionalized U.S. population (Li B. et al., 2023). For our study, we focused on individuals over 60 years of age from the NHANES 1999–2018 cycle. The NHANES study protocol received approval from the NCHS research ethics review board. Participants provided written informed consent upon enrollment. Our associated study conducted at Shangluo Central Hospital in Shangluo, China, was exempted by the institutional review board due to the use of publicly available, anonymized data, and informed consent was therefore waived (Yang et al., 2022). This study complied with the Strengthening the Reporting of Observational Studies in Epidemiology (STROBE) reporting guidelines.

### Study design and population

**FIGURE 1:**
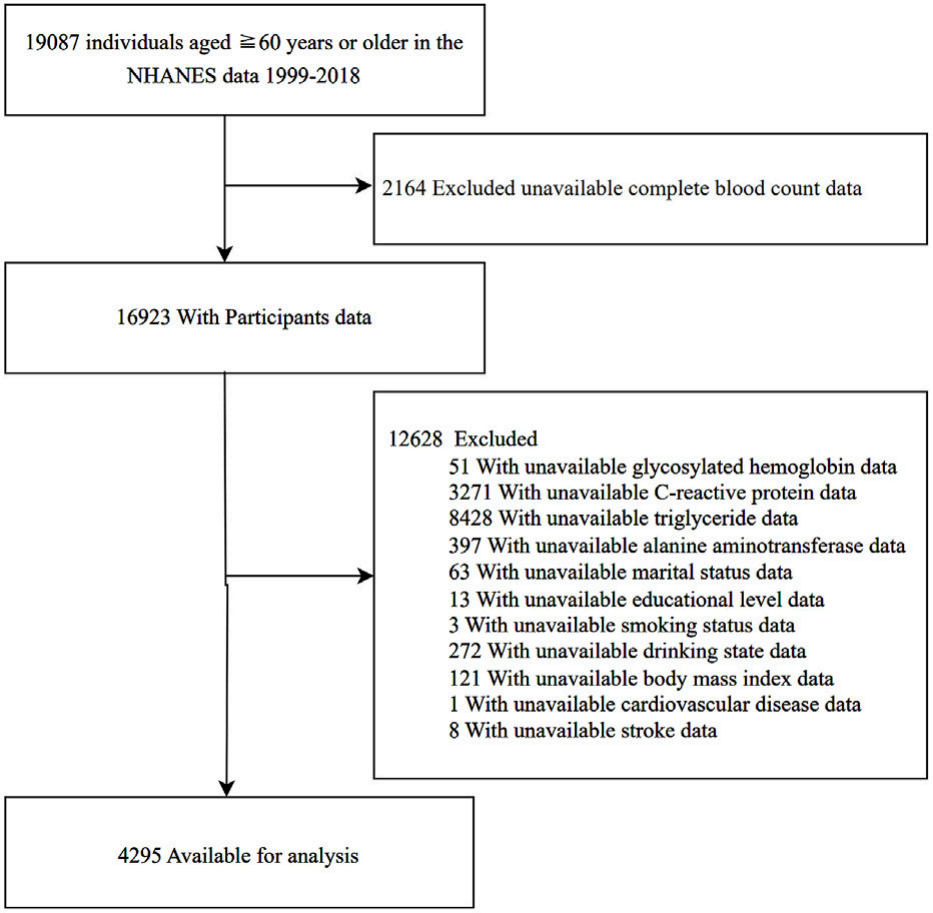
Flow diagram of the selection of eligible participants.

Data were gathered from 19,087 individuals aged 60 years or older from the NHANES database, covering the period from 1999 to 2018. We applied the following exclusion criteria: individuals with missing SII data (n = 2,164) and those missing any other covariate values (n = 12,628). Ultimately, 4,295 participants were included in our study. A flowchart of the study data selection process is presented in Figure 1.

### Definition of the systemic immune-inflammation index

Complete blood cell counts were performed on samples according to the laboratory tests listed on the NHANES website (Chen Y. et al., 2023). The SII was calculated as the product of platelet count and neutrophil count divided by the lymphocyte count, expressed in ×10^9^ cells/μl, consistent with previous studies (Hu et al., 2014).

### Determination of mortality outcomes

All-cause mortality was determined from records obtained from the National Death Index (NDI) up to 31 December 2019, which were linked to the NHANES datasets. Cause-specific mortality was assessed using the 10th edition of the International Classification of diseases (ICD-10) codes; specifically, codes C00-C97 were employed to define cancer-related deaths (Cao et al., 2023).

### Covariates

Based on prior studies, the potential covariates considered in our analysis included age, sex, and race/ethnicity (categorized as non-Hispanic White, non-Hispanic Black, Mexican American, or other). Marital status was categorized as married/living with a partner or living alone, which included individuals who were never married, separated, divorced, or widowed. Educational attainment was divided into three categories: 9, 9–12, or >12 years of education. Lifestyle characteristics such as alcohol consumption and smoking were also included. Alcohol use was defined as consuming alcohol at least 12 times per year. Individuals who smoked ≥100 cigarettes in their lifetime were categorized as smokers (Lin et al., 2022). body mass index (BMI) was calculated as body weight (kg)/height (m^2^). Weight and height were measured at the Mobile Examination Center (Liu et al., 2022). Diagnoses of asthma and emphysema were based on participants affirmatively responding to NHANES questionnaire items asking if a doctor or other health professional had ever diagnosed them with these conditions (Salo et al., 2022). Stroke and cardiovascular diseases were considered if diagnosed by a physician. Diabetes was defined based on a physician’s diagnosis, a glycosylated hemoglobin (HbA1c) level >6.5%, fasting glucose levels ≥7.0 mmol/L, blood glucose levels ≥11.1 mmol/L during a random/2-h oral glucose tolerance test, or the use of diabetes medication or insulin (ElSayed et al., 2023). The NHANES website details the procedures for the collection of blood biochemicals. Measurements included platelets, neutrophils, lymphocytes, C-reactive protein (CRP), glycated hemoglobin, albumin, triglycerides, total cholesterol, alanine aminotransferase (ALT), aspartate aminotransferase (AST), and urea nitrogen levels. These were all conducted on blood samples collected after at least 8 h of fasting, as outlined in the NHANES Detailed Laboratory Procedures (Mahemuti et al., 2023; Hong et al., 2023). The estimated glomerular filtration rate (eGFR) was calculated using the creatinine equation (Giavarina et al., 2021).

### Statistical analysis

Participant characteristics are reported as means with 95% confidence intervals (CIs) for continuous variables, and percentages with 95% CIs for categorical variables. For normally distributed data, one-way analysis of variance (ANOVA) was utilized, while the Kruskal–Wallis test was applied to data with skewed distributions. Categorical variables are presented as proportions (%), and continuous variables as either means (standard deviation [SD]) or medians (interquartile range [IQR]), as appropriate. Differences between groups were evaluated using one-way ANOVA for normally distributed data, the Kruskal–Wallis test for skewed data, and the chi-squared test for categorical variables. Multifactorial Cox regression models were employed to analyze the association between SII and the risk of all-cause mortality or cancer death, providing estimated HRs and 95% CIs. Model 1 was adjusted for sociodemographic and lifestyle characteristics (age, sex, race/ethnicity, marital status, education, smoking and alcohol consumption status), and the NHANES cycle. Model 2 included adjustments for the factors in Model 1 plus HbA1c, albumin, CRP, TG, TC, ALT, AST, Cr, BUN, and eGFR. Model 3 further adjusted for factors in Model 2 and added asthma, emphysema, cardiovascular disease, stroke, and diabetes. SII values were categorized into four subgroups based on their quartiles. Survival curves were constructed using the Kaplan-Meier method, with the log-rank test employed to assess differences in survival. Additionally, to explore the dose-response relationship between SII and mortality, we utilized restricted cubic spline (RCS) regression with a likelihood ratio test for detecting non-linearity with multivariate adjustment.

To assess the stability of the association between SII values and cancer deaths, subgroup analyses were performed based on stratification variables, employing logistic regression models and likelihood ratio tests to evaluate interactions between subgroups. *A priori* calculation of statistical power was not conducted, as the sample size was derived entirely from available data. Analyses were conducted using R version 4.2.1 (R Foundation for Statistical Computing, Vienna, Austria) and Free Statistical Software version 1.92 (Beijing Free Clinical Medical Technology Co., Ltd.). All analyses were two-sided with a P-value threshold of less than 0.05 considered statistically significant. Data analysis took place between June and July 2024.

## Results

### Study population

Of the 19,087 participants aged ≥60 years in the 1999–2018 NHANES, exclusions were made for those with missing SII data (n = 2,164) and those lacking values for other covariates (n = 12,628). Ultimately, 4,295 participants were included in the study (Figure 1).

### Baseline characteristics

The final sample consisted of 4,295 individuals aged 60 and older, with 50.3% being female and an average age of 70.7 (7.6) years. The median follow-up period was 131.7 (59.8) months, and the incidence of all-cause mortality was 50.5%. Table 1 presents the baseline characteristics of participants, categorized by quartiles of SII values. Generally, SII was higher in women, smokers, alcohol consumers, non-Hispanic whites, and those with higher educational attainment. Notably, higher SII levels were significantly associated with elevated levels of albumin, CRP, glutamine, urea nitrogen, and eGFR. Moreover, conditions such as emphysema and cardiovascular disease were also linked to increased SII values.

**TABLE 1:** General characteristics of the participants from the National Health and Nutrition Examination Survey 1999–2018 cycles.

Characteristics	Total	SII				*P*-value
		Q1	Q2	Q3	Q4	
No.	4295	1,074	1,073	1,074	1,074	
Age, years	70.7 (7.6)	69.7 (7.3)	70.0 (7.3)	70.9 (7.6)	72.4 (7.8)	<0.001
BMI, kg/m^2^	28.7 (5.8)	28.7 (5.7)	29.0 (5.6)	28.7 (5.7)	28.3 (6.1)	0.066
HbA1c, %	6.0 (1.1)	6.0 (1.2)	6.0 (1.1)	5.9 (1.1)	5.9 (1.0)	0.448
Albumin, mg/dL	4.2 (0.3)	4.2 (0.3)	4.2 (0.3)	4.2 (0.3)	4.1 (0.3)	<0.001
CRP, mg/dL	0.5 (1.0)	0.3 (0.6)	0.4 (0.5)	0.5 (0.8)	0.8 (1.6)	<0.001
TG, mg/dL	148.4 (91.4)	145.6 (110.6)	148.3 (80.4)	152.7 (90.4)	146.8 (80.9)	0.293
TC, mg/dL	201.6 (42.3)	201.0 (42.8)	203.1 (42.2)	203.3 (42.6)	199.2 (41.5)	0.080
ALT, (IU/L)	23.3 (33.2)	25.1 (17.2)	23.2 (12.0)	23.6 (61.2)	21.3 (14.7)	0.064
AST, (IU/L)	25.8 (27.6)	27.8 (14.5)	25.4 (9.6)	25.6 (50.8)	24.3 (12.4)	0.026
BUN, mg/dL	16.4 (7.2)	15.7 (6.6)	16.2 (7.0)	16.4 (7.0)	17.5 (8.1)	<0.001
eGFR, (ml/min/1.73 m^2^)	97.5 (36.7)	98.0 (34.5)	98.8 (37.9)	98.9 (37.7)	94.3 (36.7)	0.010
Gender, n (%)						0.924
Male	2,135 (49.7)	539 (50.2)	539 (50.2)	531 (49.4)	526 (49)	
Female	2,160 (50.3)	535 (49.8)	534 (49.8)	543 (50.6)	548 (51)	
Race, n (%)						<0.001
Non-Hispanic White	2,505 (58.3)	514 (47.9)	587 (54.7)	669 (62.3)	735 (68.4)	
Non-Hispanic Black	681 (15.9)	264 (24.6)	178 (16.6)	132 (12.3)	107 (10)	
Mexican American	761 (17.7)	191 (17.8)	212 (19.8)	201 (18.7)	157 (14.6)	
Others	348 (8.1)	105 (9.8)	96 (8.9)	72 (6.7)	75 (7)	
Marital status, n (%)						<0.001
Married	2,547 (59.3)	654 (60.9)	669 (62.3)	648 (60.3)	576 (53.6)	
Never married	151 (3.5)	36 (3.4)	23 (2.1)	45 (4.2)	47 (4.4)	
Living with partner	81 (1.9)	24 (2.2)	16 (1.5)	23 (2.1)	18 (1.7)	
Others	1,516 (35.3)	360 (33.5)	365 (34)	358 (33.3)	433 (40.3)	
Educational level, n (%)						0.002
<9 years	1,598 (37.2)	423 (39.4)	400 (37.3)	411 (38.3)	364 (33.9)	
9–12 years	1,022 (23.8)	214 (19.9)	281 (26.2)	244 (22.7)	283 (26.4)	
>12 years	1,675 (39.0)	437 (40.7)	392 (36.5)	419 (39)	427 (39.8)	
Smoking, n (%)	2,314 (53.9)	526 (49)	580 (54.1)	592 (55.1)	616 (57.4)	<0.001
Drinking, n (%)	3,520 (82.0)	872 (81.2)	879 (81.9)	876 (81.6)	893 (83.1)	0.667
Asthma, n (%)	439 (10.2)	95 (8.8)	108 (10.1)	119 (11.1)	117 (10.9)	0.303
Emphysema, n (%)	186 (4.3)	32 (3)	29 (2.7)	47 (4.4)	78 (7.3)	<0.001
CVD, n (%)	1,029 (24.0)	244 (22.7)	234 (21.8)	245 (22.8)	306 (28.5)	<0.001
Stroke, n (%)	345 (8.0)	79 (7.4)	85 (7.9)	80 (7.4)	101 (9.4)	0.271
Diabetes, n (%)	1,345 (31.3)	327 (30.4)	349 (32.5)	318 (29.6)	351 (32.7)	0.322
Status, n (%)						<0.001
Alive	2,124 (49.5)	633 (58.9)	560 (52.2)	531 (49.4)	400 (37.2)	
Death	2,171 (50.5)	441 (41.1)	513 (47.8)	543 (50.6)	674 (62.8)	

SII, systemic immune inflammatory index; BMI, body mass index; HbA1c, glycated hemoglobin; CRP, C-reactive protein; TG, triglyceride; TC, total cholesterol; ALT, alanine aminotransferase; AST, aspartate aminotransferase; BUN, blood urea nitrogen; eGFR, estimated glomerular filtration rate; CVD, cardiovascular diseases.

Note: The continuous data were shown as mean (standard deviation, SD), and differences between groups were compared using a T-test, one-way analyses of variance (normal distribution), and Kruskal–Wallis tests (skewed distribution). The categorical data were shown as numbers and percentages [n (%)], and differences between groups were compared using the Chi-squared test.

### Associations between SII and mortality

**FIGURE 2:**
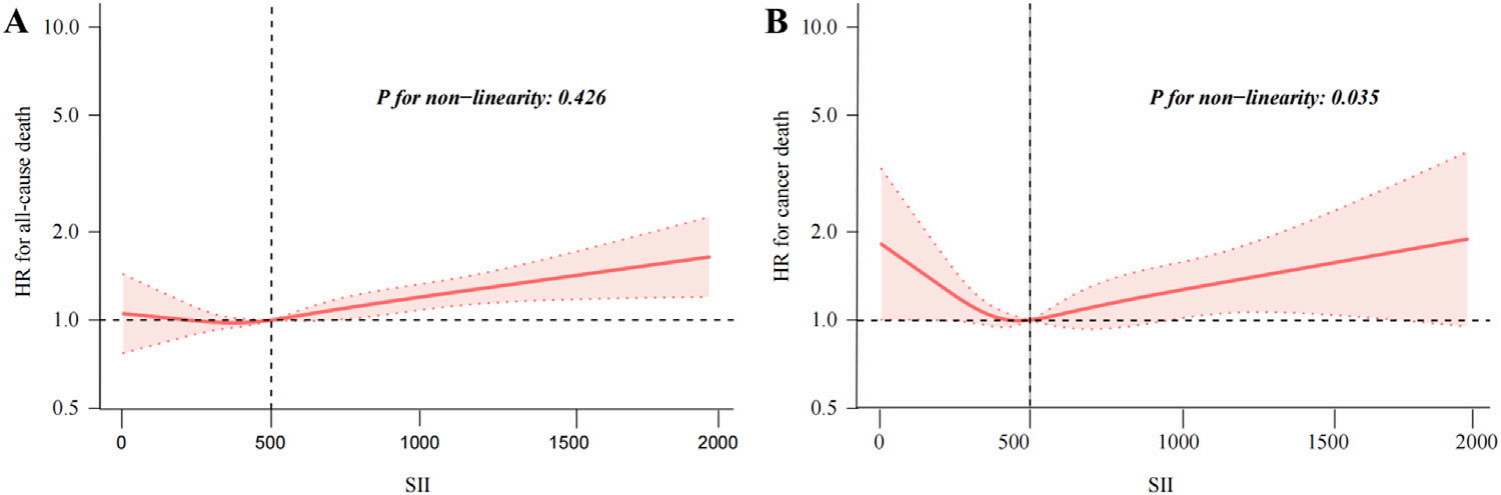
Restricted cubic spline fitting for the association between SII with mortality. Association of SII levels with the all-cause **(A)**, and cancer mortality **(B)**.

**FIGURE 3:**
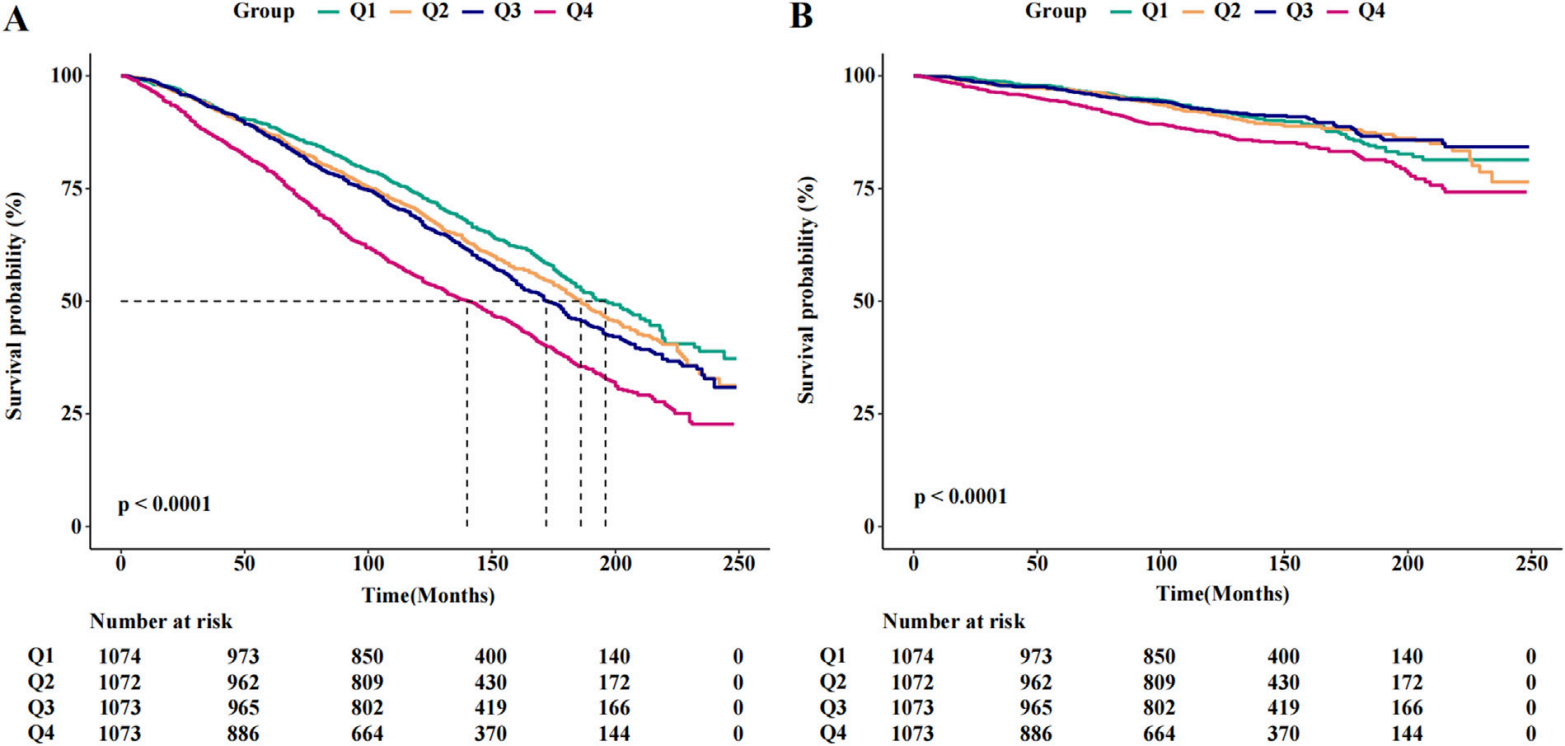
The Kaplan-Meier curves for 20-year occurrence of **(A)** all-cause and **(B)** cancer mortality, by quartiles of SII.

The results of the Cox regression models, as presented in Table 2, indicate that SII was significantly associated with the risk of all-cause mortality (HR = 1.73, 95% CI = 1.54–1.95) in the unadjusted model. This association remained robust and statistically significant after multivariate adjustments in model 1 (HR = 1.40, 95% CI = 1.24–1.59), model 2 (HR = 1.31, 95% CI = 1.16–1.49), and model 3 (HR = 1.31, 95% CI = 1.15–1.48). In contrast, the association between SII and cancer death, though significant in the unadjusted model (HR = 1.52, 95% CI = 1.19–1.95), showed reduced stability and was not statistically significant across the adjusted models: model 1 (HR = 1.31, 95% CI = 1.02–1.68), model 2 (HR = 1.24, 95% CI = 0.96–1.60), and model 3 (HR = 1.23, 95% CI = 0.95–1.59). The RCS results revealed a linear relationship between SII and all-cause mortality, with increasing SII values correlating with higher mortality risk, whereas a U-shaped relationship was observed between SII and cancer mortality (Figures 2A, B). Threshold analysis indicated that the HR for the risk of cancer death for participants with an SII <504.52 was 0.998 (95% CI: 0.997 to 0.999, P = 0.037) (Table 3). For those with an SII ≥504.52, the HR was 1.004 (95% CI: 1.002 to 1.007, P = 0.003), suggesting that a higher SII was associated with an increased risk of cancer death. Survival curves indicated that patients with an SII >725.33 had a worse prognosis compared to other groups during the follow-up period, P < 0.001 on the log-rank test) (Figures 3A, B).

**TABLE 2:** HRs (95% Cis) for all-cause and cancer mortality based on SII quartiles in American 60 years and older.

	Q1	Q2	Q3	Q4	P trend
Levels of SII	<359.60	359.60–508.89	508.89–725.33	>725.33	
AII-cause mortality					
No. Deaths/total	441/1074	513/1073	543/1074	674/1074	
Crude	Reference	1.14 (1.01∼1.30) 0.04	1.23 (1.09∼1.40)	1.73 (1.54∼1.95)	0.001
Model 1	Reference	1.15 (1.01∼1.30)	1.13 (0.99∼1.28)	1.40 (1.24∼1.59)	0.001
Model 2	Reference	1.15 (1.01∼1.31)	1.11 (0.98∼1.26)	1.31 (1.16∼1.49)	0.001
Model 3	Reference	1.16 (1.02∼1.33)	1.13 (0.99∼1.28)	1.31 (1.15∼1.48)	0.001
Cancer Mortality					
No. deaths/total	110/1074	113/1073	98/1074	148/1074	
Crude	Reference	1.01 (0.78∼1.32)	0.89 (0.68∼1.18)	1.52 (1.19∼1.95)	0.003
Model 1	Reference	1.00 (0.77∼1.30)	0.83 (0.63∼1.10)	1.31 (1.02∼1.68)	0.084
Model 2	Reference	1.02 (0.78∼1.33)	0.83 (0.63∼1.09)	1.24 (0.96∼1.60)	0.227
Model 3	Reference	1.03 (0.79∼1.34)	0.83 (0.63∼1.10)	1.23 (0.95∼1.59)	0.247

HR (95% CI), hazard ratios (HRs) with their 95% confidence intervals (CIs); Q, quartiles; SII, systemic immune inflammatory index; BMI, body mass index; HbA1c, glycated haemoglobin; CRP, C-reactive protein; TG, triglyceride; TC, total cholesterol; ALT, alanine aminotransferase; AST, aspartate aminotransferase; BUN, blood urea nitrogen; eGFR, estimated glomerular filtration rate; CVD, cardiovascular diseases.

Model 1 adjusted for demographic social factors (age, genders, race/ethnicity, marital status, education level, smoking status, drinking state, and BMI.).

Model 2 was adjusted for model 1 + HbA1c, albumin, CRP, TG, TC, ALT, AST, BUN, and eGFR.

Model 3 was adjusted for model 2 + asthma, emphysema, CVD, stroke, and diabetes.

**TABLE 3:** Association between SII and cancer mortality using two-piecewise regression models.

SII	Adjusted model	
	HR (95% CI)	P-value
<504.52	0.998 (0.997–0.999)	0.037
≥504.52	1.004 (1.002–1.007)	0.003
Log-likelihood ratio test		0.032

HR (95% CI), hazard ratios (HRs) with their 95% confidence intervals (CIs); SII, systemic immune inflammatory index; BMI, body mass index; HbA1c, glycated haemoglobin; CRP, C-reactive protein; TG, triglyceride; TC, total cholesterol; ALT, alanine aminotransferase; AST, aspartate aminotransferase; BUN, blood urea nitrogen; eGFR, estimated glomerular filtration rate; CVD, cardiovascular diseases.

Adjusted for age, genders, race/ethnicity, marital status, education level, smoking status, drinking state, and BMI, HbA1c, albumin, CRP, TG, TC, ALT, AST, BUN, eGFR, asthma, emphysema; CVD, stroke, and diabetes.

### Stratified analyses based on other variables

Subgroup analysis, after adjusting for age, sex, body mass index, CRP, creatinine clearance, and diabetes, revealed an interaction between SII and body mass index in the subgroup analysis of SII and all-cause mortality (p < 0.05). In the subgroup analysis of SII and cancer mortality, the relationship between SII and cancer mortality was stable, with no significant interaction between subgroups (p > 0.05), and the difference was not statistically significant (Supplementary Figure A, B)

## Discussion

The results of the study showed an association between SII and the risk of all-cause and cancer mortality even after adjustment for multifactorial modelling. SII was linearly associated with all-cause mortality and U-shaped with cancer mortality, and survival curve analyses showed an increased risk of all-cause and cancer deaths in the group with higher levels of SII (> 725.33). In addition, we found that in the subgroup analysis of SII with all-cause mortality, the results showed an interaction between SII and body mass index (P < 0.05). This may be related to the higher risk of dying from cardiovascular disease in individuals with higher body mass index. However, in the subgroup analysis of SII and cancer mortality, there was no interaction between subgroups (P > 0.05).

Previous studies have reported significant associations between SII and the prognosis of chronic diseases and cancer (Ye et al., 2023; Tian et al., 2022; Nakamoto et al., 2023). Among Americans with hypertension, cardiovascular disease, type 2 diabetes, and adult frailty, SII demonstrated a U-shaped relationship with both all-cause and cancer mortality. When SII was treated as a categorical variable, it was consistently associated with all-cause mortality after multi-model adjustment. Nevertheless, the relationship between SII and cancer mortality varied. SII was significantly associated with cancer mortality in the cardiovascular disease and frailty populations (Xiao et al., 2023; Zheng et al., 2023). In the hypertension and type 2 diabetes populations, SII was not significantly associated with the risk of cancer mortality (Cao et al., 2023; Chen C. et al., 2023). Additionally, SII showed a J-shaped relationship with all-cause mortality in patients with non - alcoholic fatty liver disease (NAFLD) and osteoarthritis (Zhou et al., 2024; Zhao et al., 2023), and a linear rather than U-shaped relationship in the sarcopenia and chronic renal failure populations (Zeng et al., 2024; Tang et al., 2024). These studies have shown that the relationship between SII and the risk of all-cause mortality and cancer mortality varies between populations. In our study, we found a linear relationship between elevated SII and all-cause mortality and a U-shaped relationship between SII and cancer mortality in the US population aged 60 years and older. This adds to our understanding of the relationship between SII and mortality risk in populations.

To further investigate the association between SII and cancer mortality, we explored a number of factors that influence the association with cancer mortality, such as female, age, higher body mass index, higher CRP, creatinine clearance, and diabetes. Consistent with previous studies, we found an association between SII and cancer mortality in female participants, which may be attributed to the higher risk of breast, uterine and ovarian cancers in this group (Zhou et al., 2023; Extermann et al. 2023; Huang et al. 2019; Mao et al. 2023). In older populations, there is an association between SII and mortality risk, which may be related to chronic inflammation, impaired immune function and increased cancer risk ( Li et al., 2021). In obese populations, SII is positively correlated with the percentage of body fat distribution in obese individuals (body mass index >30 kg/m^2^), and overweight and obesity may increase the risk of all-cause and cancer mortality (Liu et al., 2024; Molina-Montes et al., 2021). Both SII and CRP can be used to reflect the body’s inflammation level, and clinical studies have found that higher CRP potentially increases the risk of cancer mortality (Wulaningsih et al., 2016). Previous studies have shown that the relationship between SII and creatinine clearance and cancer mortality is unclear. (Zhai et al., 2024) found that the estimated glomerular filtration rate (eGFR) tended to be lower in patients with a high SII group in immunoglobulin A (IgA) nephropathy, and that elevated SII was associated with a poor prognosis in diabetic nephropathy and IgA nephropathy (Guo et al., 2022). In contrast, chemotherapy, radiotherapy, and targeted therapies can cause acute kidney injury with decreased creatinine clearance, which increases the risk of cancer mortality (Jagieła et al., 2021). In diabetic populations, elevated SII is positively associated with diabetes (Nie et al., 2023), and previous studies have confirmed the association between diabetes and the risk of cancer mortality (Pearson-Stuttard et al., 2021; Yeh et al., 2018).

Low-grade systemic inflammation is a characteristic of aging, with a significant correlation between chronic inflammation and cardiovascular, metabolic, and cancer diseases (Vasto et al., 2009). The morbidity and mortality of most chronic diseases increase with age, particularly in individuals over 60 years old (Prince et al., 2015). The SII is used to evaluate the body’s immune level and inflammatory status through the ratio of neutrophils and platelets to lymphocytes (Hang et al., 2022). Neutrophils play a crucial role in maintaining immune function and in the pathogenesis of diseases. The elimination of pathogenic microorganisms, mediation of tissue damage, and aseptic inflammation are mediated by the release of neutrophil extracellular traps (NETs). Simultaneously, NETs can interact with P-selectin to activate platelets, promote thrombosis, and lead to organ damage (Hidalgo et al., 2022). Moreover, neutrophil extracellular traps can induce the production of autoimmune complexes that exacerbate autoimmune diseases and participate in the inflammatory response to induce tumors and promote cancer progression (Mutua et al., 2021). Lymphocytes, classified into T-lymphocytes, B-lymphocytes, and natural killer cells, maintain the body’s immune function through cellular and humoral immunity (Yazicioglu et al., 2024). Activated lymphocytes can also cause tissue damage and organ dysfunction by releasing mitochondrial danger-associated molecular patterns (DAMPs), which mediate the inflammatory response to pathogens (Faas et al., 2020). The heterogeneity, differentiation, and proliferation of lymphocytes are relevant to the development of infectious diseases, chronic inflammation, tumors, and autoimmune diseases (Sun et al., 2023; Chapman et al., 2022). Platelets are not only essential for hemostasis but are also intricately involved in the body’s inflammatory response, thrombosis, cancer occurrence, and metastasis. They also interact with neutrophils and lymphocytes and participate in the systemic immune-inflammatory response (Mandel et al., 2022). In the elderly population (especially those with chronic comorbidities), high levels of inflammation are commonly observed. Meanwhile, the immune system undergoes immune senescence with age, and immune cell senescence acquires an inflammatory senescence - associated secretory phenotype (SASP), which further induces immune cell senescence and immune deterioration, leading to an imbalance in the body’s immune-inflammation levels (Karanth et al., 2021; Saavedra et al., 2023). Inflammation and immune aging cause an increase in immune- inflammation levels. High SII promotes the development of chronic inflammation and cancer and is closely related to the poor prognosis of many diseases (Benz et al., 2022; Zhang et al., 2023; Liu et al., 2023).

The current study has some limitations. First, while the analysis utilized a large sample from NHANES and accounted for relevant covariates, the study population was restricted to individuals aged 60 years or older in the United States, which limits its generalizability to other demographics. Second, the SII results were based on a single measurement; the absence of data from multiple repeated measurements might not accurately represent the long-term immune-inflammatory status, potentially underestimating the association. Third, the reliance on self-reported data for conditions like asthma, emphysema, and stroke could introduce memory bias. Finally, given the observational nature of our study, we could not establish a causal relationship between SII and mortality outcomes.

## Conclusion

There is a significant association between higher SII levels and increased risk of all-cause and cancer-specific mortality in the US population aged 60 years and older.

## Data Availability

These survey data are free and publicly available, and can be downloaded directly from the NHANES website (https://wwwn.cdc.gov/nchs/nhanes/default.aspx) by users and researchers worldwide.
