# Supplementary material for: Association of systemic immune-inflammatory index with all-cause and cancer mortality in Americans aged 60 years and older
Source: Front Aging. 2025 Mar 10;6:1502746. doi: 10.3389/fragi.2025.1502746 (PMC11931307; doi:10.3389/fragi.2025.1502746)
Supplement: Supplementary file 1 [file Table1.docx]

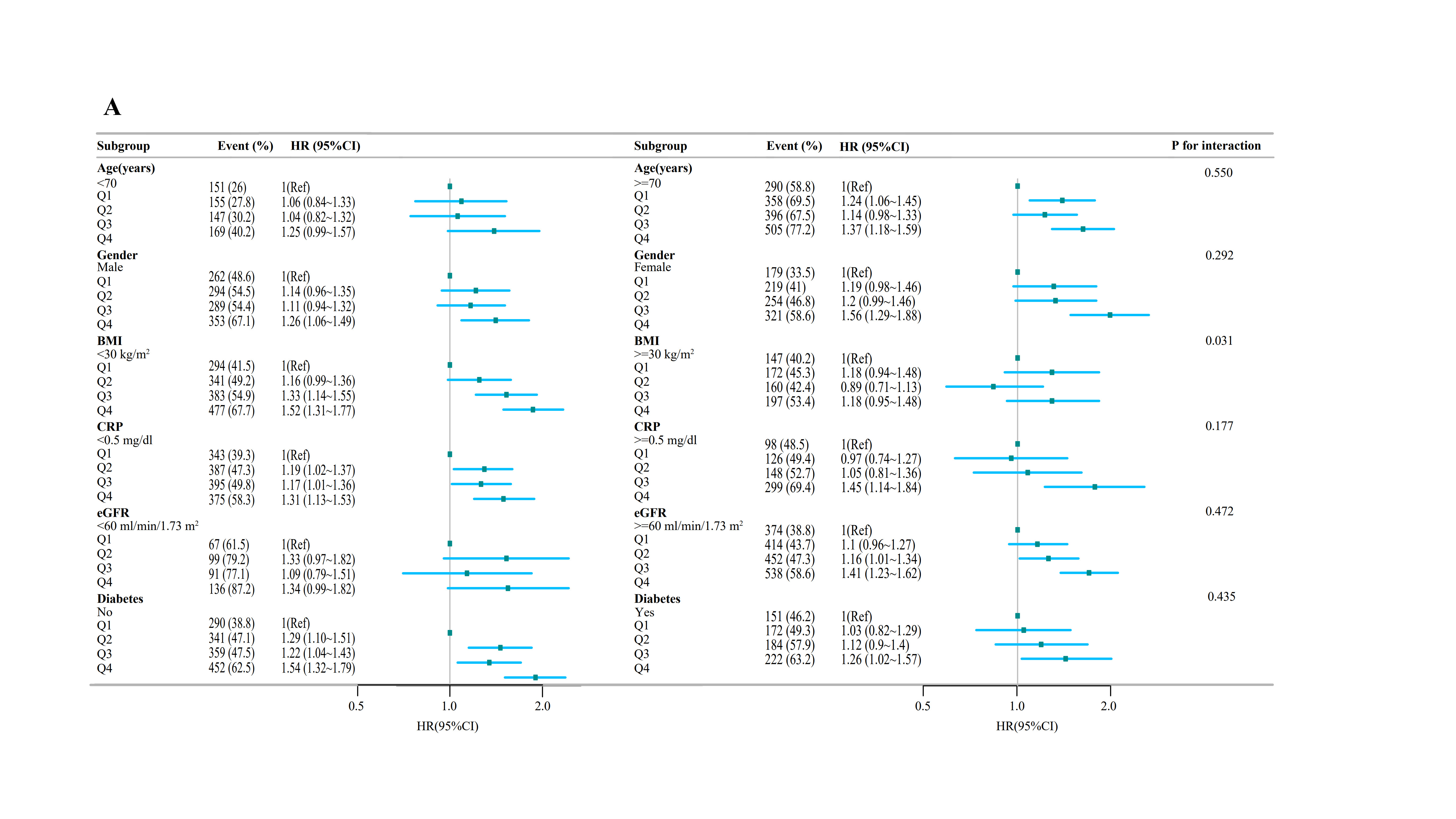


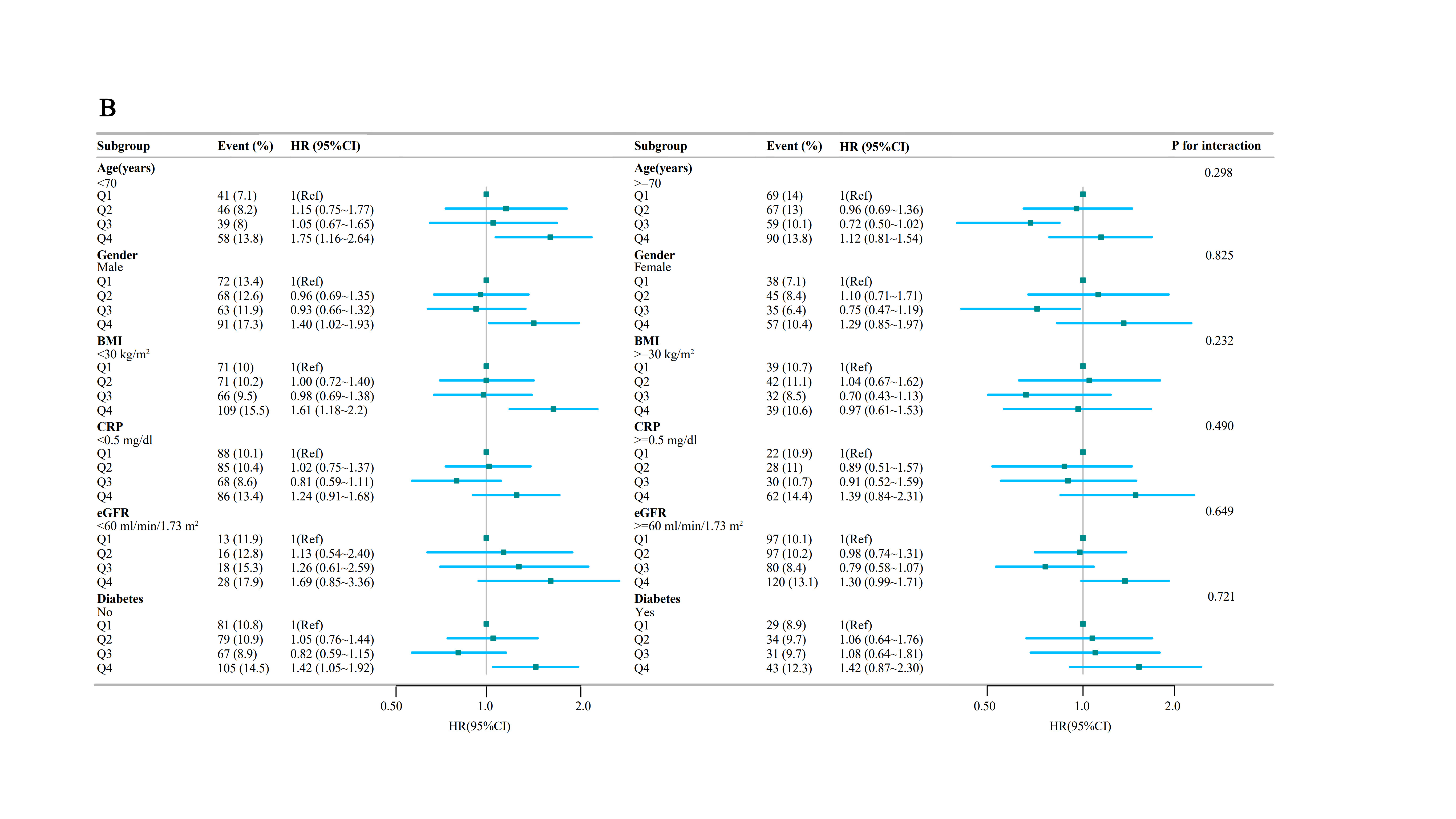


Forest plot of subgroup analyses of the association of SII with all-cause mortality（A） and cancer mortality(B).

Mortality. Hazard ratios (HRs) were calculated from multivariate Cox regression models adjusted for the variables listed in the fully adjusted model, except for those used for stratification.
